# Effects of transcutaneous spinal stimulation with gait training on walking-related outcomes in stroke survivors: a systematic review

**DOI:** 10.1007/s10072-026-08822-x

**Published:** 2026-01-31

**Authors:** Jibrin S. Usman, Thomson W. L. Wong, Shamay S. M. Ng

**Affiliations:** Department of Rehabilitation Sciences, The Hong Kong Polytechnic University, the Hong Kong Special Administrative Region of the People’s Republic of China, Hong Kong, China

**Keywords:** Gait training, Spinal stimulation, Stroke survivors, Walking-related outcomes

## Abstract

**Background:**

Stroke survivors present with various deficits, and gait training has been reported to have a positive impact on stroke survivors. Transcutaneous spinal stimulation (TSS; e.g., tsDCS, tSCS, and phasic TSS) is an emerging technique for post-stroke recovery.

**Objectives:**

This systematic review evaluated the scientific evidence on the effects of gait training (GT) combined with transcutaneous spinal stimulation (TSS) on walking and related outcomes in stroke survivors.

**Methods:**

In this systematic review, the EMBASE, PubMed, PEDro, Cochrane Library, and Web of Science databases were searched from their inception to November 2025. Randomised controlled trials were included, and their methodological quality and risk of bias (ROB) were evaluated using the PEDro scale and Cochrane ROB assessment tool, respectively. Qualitative and quantitative syntheses were used for the data analysis.

**Results:**

Cathodal transcutaneous spinal stimulation combined with gait training probably improves primary gait outcomes (walking capacity, cadence, paretic lower limb strength, and walking speed) with moderate certainty, while exhibiting little or no difference in secondary gait outcomes. In contrast, anodal tsDCS showed variable/mixed effects on gait outcomes.

**Conclusions:**

Moderate-certainty evidence shows that cathodal transcutaneous spinal stimulation combined with gait training probably improves primary walking outcomes in stroke survivors.

**Supplementary Information:**

The online version contains supplementary material available at 10.1007/s10072-026-08822-x.

## Introduction

Stroke survivors present with persistent abnormalities in gait throughout the chronic stage of the stroke [1], and the gait limitation is a main contributing factor to post-stroke functional disability [2]. Stroke survivors also present with various neurological problems such as motor, cognitive and sensory, with impairment in motor function negatively impacting their walking [3]. The broad spectrum and hierarchical nature of gait impairments in post-stroke hemiplegia reflect the mechanical consequences of muscle weakness, spasticity, abnormal synergies, and their interactions [4]. The predominant gait pattern observed following stroke is the hemiparetic gait, characterised by asymmetric spatiotemporal and kinematic gait features [3]. Stroke survivors show longer nonparetic stance and paretic swing time, reduced nonparetic swing, and extended double stance phases, particularly in those with low gait speed, with marked stance/swing asymmetry observed [5].

One of the main goals of stroke survivors is gaining independence in walking [6]. The beneficial effects of gait training techniques, such as robot-assisted gait training [7] and treadmill training [8], on walking function in stroke survivors have been reported. Moreover, Physical exercise has been linked to protective impacts to the ageing brain of older individuals [9]. Although rehabilitation is aimed at gait improvement, locomotor recovery is usually incomplete; thus, the development of strategies to enhance the beneficial effects of training and to aid locomotor learning is vital [10]. Physical activity and exercise have an obvious impact on neuroplastic changes in those with neurological conditions such as stroke, PD, SCI, and TBI, and combining neuromodulation with exercise provides the possibility to enhance such neuroplastic impacts, which is an interesting developing field in individualised rehabilitation [11]. In Mammals, spinal cord neural networks have been found to be involved in the control of postural and locomotor functions and can produce walking pattern when there is no supraspinal influence and peripheral afferent input [12].

Recently, it has been shown that, besides playing a role in the acquisition and maintenance of new motor skills, the spinal cord is also involved in modulating of cognitive and motor functions reliant on cortical motor areas [13]. Spinal cord stimulation is an emerging neuromodulation approach that can be used to address upper and lower extremity hemiparesis [14]. There are several types of spinal cord stimulation, such as epidural; and transcutaneous [consisting of transcutaneous electrical spinal cord stimulation (tSCS) and transcutaneous spinal direct current stimulation (tsDCS)] [14]. The application of a homologous direct current to the spinal cord, such as tsDCS, is a technique similar to transcranial direct current stimulation (tDCS) [15]. tsDCS, a promising non-invasive neuromodulation method, delivers a weak direct current via pair of skin electrodes, to modulate the spinal cord activity through an induced electric field [16]. It is a safe and convenient neuromodulation technique, and the knowledge of its impact on the somatosensory cortex is still uncommon [15]. However, it has been reported that it could probably influence locomotor training [10].

tsDCS, a potential adjunctive technique for post-stroke recovery, induces local and cortical neuroplastic changes and exerts activation in cortical and corticospinal pathways with the aid of neurophysiological techniques in humans [13]. tsDCS, through its supraspinal effects, might be appropriate for cognitive and motor recovery in stroke survivors [13].

Based on the above-mentioned studies, and to the best of our knowledge, this is the first systematic review of the effects of gait training combined with TSS (e.g., tsDCS, tSCS, and phasic TSS) on walking related outcomes in stroke survivors. Accordingly, this systematic review aimed to determine the scientific evidence of the effects of gait training combined with transcutaneous spinal stimulation (TSS; e.g. tsDCS, tSCS, and phasic TSS) on walking-related outcomes in stroke survivors.

### Methods

The review was registered with PROSPERO (registration number: CRD42024558037) and conducted following the Preferred Reporting Items for Systematic Reviews and Meta-Analyses (PRISMA) guidelines checklist. The PICOS method was employed to form the research question: what is the effect of active TSS (alone or with other adjuvant stimulation) combined with gait training (I = intervention) on walking-related outcomes (O = outcome) in stroke survivors (P = population), when compared with sham TSS/active variant (alone or with other adjuvant stimulation) combined with gait training or gait training alone (C = comparison) in randomised controlled trials (RCTs) (S = study design)? Following the methodologies delineated in our prior reviews [17, 18], we carried out the procedure for searching articles, methodological quality and risk of bias evaluations, selection of studies, and extraction of data. Refinements were made to the eligibility criteria, searched databases, search dates, extracted data, and search strategy according to the aims of this systematic review.

#### Search of articles

The EMBASE, PubMed, PEDro, Cochrane Library, and Web of Science databases were searched from their inception to November 2025. The PICOS technique was used to develop the search strategy. All databases were searched based on their requirements. The details of the search strategy used in most databases are provided in the Supplementary Material. A manual search of the reference lists of the included studies and reviews was also conducted. Studies obtained from the general literature search were involved in the review. The results were transferred to the EndNote reference manager, where duplicates were removed, and the remaining results were further screened for titles, abstracts, and full texts. The search was independently conducted by one of the reviewers (J.S.U.) and verified by the other two reviewers (T.W.L.W. and S.S.M.N.).

#### Selection criteria

The criteria for including studies were as follows: (i) studies conducted with human stroke survivors; (ii) studies reporting the effects of TSS (e.g. tsDCS, tSCS, and phasic TSS) combined with gait training; (iii) full-text RCTs, either parallel or crossover; and (iv) studies published in English in peer-reviewed journals. Abstracts from conferences, theses, and review studies were excluded.

#### Study selection and extraction of data

After duplicates were removed, the titles and abstracts of the remaining studies were independently screened by two reviewers (J.S.U. and T.W.L.W.) based on the eligibility criteria. In cases where the two reviewers (J.S.U. and T.W.L.W.) could not agree on the inclusion of a study, the third reviewer (S.S.M.N.) was consulted to resolve the disagreement. Thereafter, the full texts of the eligible studies were obtained for the full-text screening phase. The data extracted from each of the included studies comprised the study design, sample size, sex, age, author name and publication year, clinical characteristics of the population (chronicity, stroke duration, type of stroke, affected side), intervention group (s), outcomes, type of training combined with spinal stimulation, evaluation periods, stimulation parameters (type of stimulation, location, intensity, duration, electrode size, number of sessions, adverse effects), and results/findings and conclusions. The extracted data were recorded in Microsoft Excel.

#### Methodological quality and risk of bias evaluation

The methodological quality (MQ) of the studies included in the review was evaluated using the PEDro scale. This 11-point scale assesses the MQ of studies [19]. The 11 items address the study’s internal validity and statistical reporting; the first item deals with the eligibility criteria and is not considered in the overall score calculation [20]. The remaining 10 items deal with internal validity and are scored as 0 or 1, representing No (absent) and Yes (present), respectively [20]. The total score obtained was then categorised as follows: 0–3 (poor quality), 4–5 (fair quality), 6–8 (good quality), and 9–10 (excellent quality) [21, 22].

The risk of bias (ROB) in the studies included in the review was evaluated using the Cochrane tool for assessing the ROB in RCTs (Figs. 2a and b). Decisions on whether the ROB was unclear, high, or low were made for each item within the following domains: selection bias (random sequence generation and allocation concealment), reporting bias (selective reporting), performance bias (blinding of personnel and participants), detection bias (blinding of outcome assessment), attrition bias (incomplete data), and other biases (other sources of bias) [23]. Each study received a general score based on the overall ratings across all domains and corresponding decisions (unclear, high, or low). The evaluation was conducted independently by two reviewers (J.S.U. and T.W.L.W.). In cases where an agreement could not be reached during the evaluation process, a third reviewer (S.S.M.N) was consulted to resolve the disagreement.

#### Data analysis

Both qualitative and quantitative synthesis methods were used for data analysis. Qualitative synthesis involved describing the MQ, ROB, and characteristics of the studies included in the review.

## Results

### Qualitative synthesis of the results

#### Eligible studies selection

**Fig. 1:**
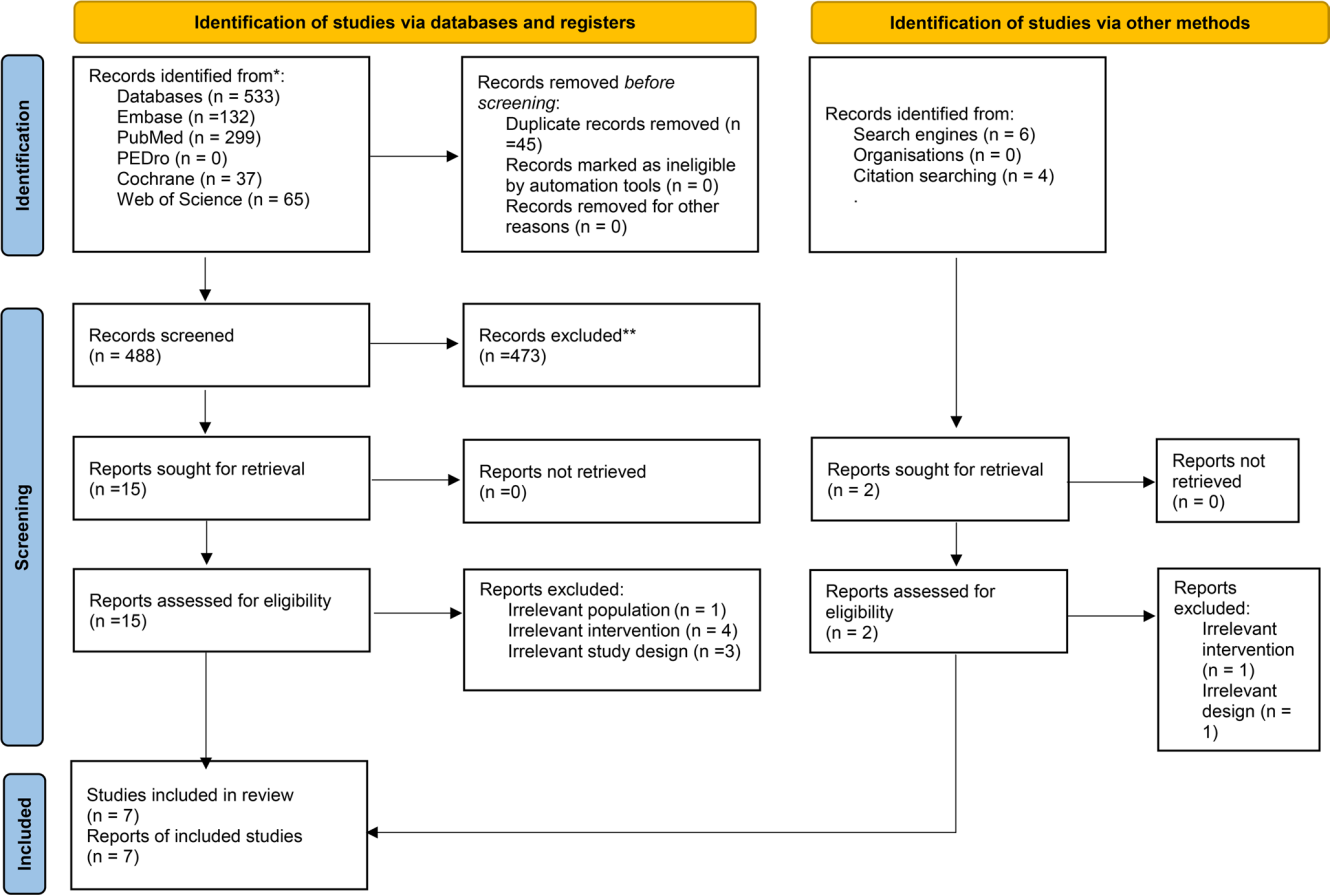
PRISMA flowchart of the review process

The search of the databases and other sources yielded 543 studies. After removing 45 duplicates, 498 studies remained. During the title and abstract screening stage, 481 studies were not included for not meeting the review criteria. Subsequently, the full texts of the remaining 17 studies were assessed based on the review criteria. Seven studies were found to be eligible and included in the review. All of the included studies were RCTs (six parallel and one crossover). Figure 1 provides details of the study identification and selection process in accordance with the PRISMA guidelines.

#### Methodological quality of the eligible studies

Of the seven studies, four were rated as having excellent and three as having good MQ. Regarding the PEDro scale domains, all of the included studies specified the eligibility criteria, used random allocation, ensured that groups/conditions were similar at baseline in terms of important prognostic indicators, measured key outcomes from the majority of participants initially allocated to the groups, reported between-group comparisons, and provided point measures and measures of variability for key outcomes. In four of the included studies, allocation concealment, subject blinding, and outcome assessor blinding were implemented. In all but one of the studies, there was no blinding of experimenters, and all subjects for whom outcome measures were available received the intervention as allocated in all but one of the studies (Table 1).

**Table 1:** Methodological quality of the included studies based on PEDro scale criteria

Study/Author (year)	Item 1	Item 2	Item 3	Item 4	Item 5	Item 6	Item 7	Item 8	Item 9	Item 10	Item 11	Total Score	Overall Quality assessment
Awosika (2020)	Yes	Yes	Yes	Yes	Yes	Yes	Yes	Yes	No	Yes	Yes	9	Excellent quality
Moshonkina (2022)	Yes	Yes	No	Yes	No	No	No	Yes	Yes	Yes	Yes	6	Good quality
Park (2025)	Yes	Yes	No	Yes	No	No	No	Yes	Yes	Yes	Yes	6	Good quality
Picelli (2015)	Yes	Yes	Yes	Yes	Yes	No	Yes	Yes	Yes	Yes	Yes	9	Excellent quality
Picelli (2018)	Yes	Yes	Yes	Yes	Yes	No	Yes	Yes	Yes	Yes	Yes	9	Excellent quality
Picelli (2019)	Yes	Yes	Yes	Yes	Yes	No	Yes	Yes	Yes	Yes	Yes	9	Excellent quality
Tani (2025)	Yes	Yes	No	Yes	No	No	No	Yes	Yes	Yes	Yes	6	Good quality

**Item 1**: eligibility criteria specified, **Item 2**: subjects were randomly allocated to groups(in a crossover study, subjects were randomly allocated an order in which treatments were received), **Item 3**: Allocation was concealed, **Item 4**: the groups were similar at baseline regarding the most important prognostic indicators, **Item 5**: there was blinding of all subjects, **Item 6**: there was blinding of all therapists who administered the therapy, **Item 7**: there was blinding of all assessors who measured at least one key outcome, **Item 8**: Measures of at least one key outcome were obtained from more than 85% of the subjects initially allocated to groups, **Item 9**: all subjects for whom outcome measures were available received the treatment or control condition as allocated or, where this was not the case, data for at least one key outcome was analyzed by “intention to treat”, **Item 10**: the results of between-group statistical comparisons are reported for at least one key outcome, **Item 11**: The study provides both point measures and measures of variability for at least one key outcome

#### Risk of bias in eligible studies

**Fig. 2:**
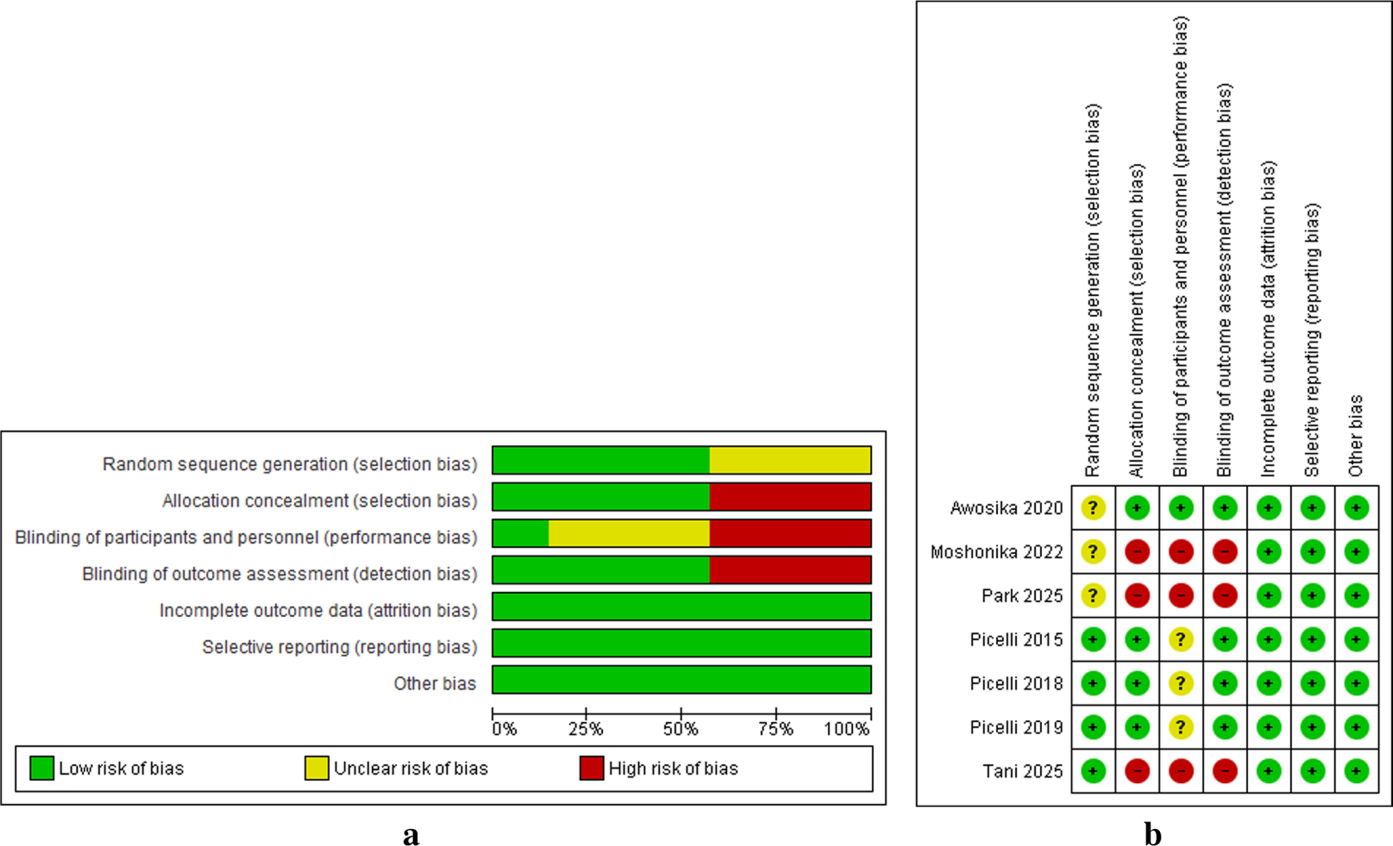
**a** Risk of bias graph for the included studies. **b** risk of bias summary for the included studies

All studies demonstrated a low ROB in the reporting, attrition, and other bias domains. In the domains of detection and selection bias (allocation concealment), four studies had a low ROB, whereas the other three had a high ROB. In terms of performance bias, one study had a low ROB, three had an unclear ROB, and three had a high ROB. In the domain of selection bias (random sequence generation), four studies had a low ROB and three had an unclear ROB (Fig. 2a and b).

#### Characteristics of the eligible studies

All of the studies involved chronic stroke survivors; six studies used a parallel RCT design, while one used a crossover design. Four studies focused on tsDCS, while two [12, 24] investigated tSCS. In total, 171 chronic stroke survivors participated in the studies (110 males and 61 females). The mean age of the participants across the studies ranged from 53.33 to 64.75 years. In all seven studies, spinal stimulation was combined with gait training (e.g., treadmill and robot-assisted training) [12, 24–29]. All six parallel RCTs applied interventions for multiple sessions/weeks, whereas the only crossover RCT [29] applied a short-term single session/day intervention. All included studies assessed outcomes at baseline and after the intervention, with three studies [25, 27, 28] also including additional follow-up evaluations (Table 2).

**Table 2:** Characteristics of the included studies

Author (Year)	Design	*N* Gender	Age (years) Mean ± SD	Clinical Characteristics of the patient population	Intervention(s)/Groups	Outcomes	Types of training combined with tsDCS Evaluation period
Awosika (2020)	RCT-P	30 16 M,14 F	EG: 58.55 ± 7.61 CG: 54.74 ± 10.90	Chronicity: Chronic Stroke duration: EG:62.22(66.3); CG: 62.25 (72.0) Stroke type: IS = 24; HM = 6 Side affected: *R* = 18; L = 12	EG: BLTT + a-tsDCS CG: BLTT + Sham tsDCS	Walking speed (GS), cadence, stride length, and walking capacity	BLTT (motor) Pre, post & 2 weeks follow up
Moshonkina (2022)	RCT-P	20 15 M, 5 F	EG: 56.4 ± 9.0 CG: 52.9 ± 10.8	Chronicity: Chronic Stroke duration (M): EG:7.1(3.0); CG: 6.9 (3.5) Stroke type: IS = 17; HM = 3 Side affected: R = NR; L = NR	EG: tSCS during walking/stepping (on a treadmill and the floor) + standard rehabilitation therapy CG: sham tSCS during stepping/walking (on a treadmill and the floor) + standard rehabilitation therapy	Primary outcomes: Walking performance [walking speed (10-m walk test), walking capacity (6-min walk test). Secondary outcomes: motor impairment/control, functional independence, muscle strength, muscle tone, balance, spatiotemporal and kinematic parameters.	Standard rehabilitation therapy and stepping/walking on a treadmill and the floor (motor) before and after
Park (2025)	RCT-CR	11 5 M, 6 F	61.73 ± 8.8	Chronicity: Chronic Stroke duration (Y): 6.36 ± 4.69 Diagnosis: Single unilateral, supratentorial stroke of either ischemic or haemorrhagic aetiology Side affected: *R* = 5; L = 6	ECN: TSS + VF + locomotor training on a DIT CCN: sham TSS + VF + locomotor training on a DIT	Vertical ground reaction force (vGRF), muscle activity (EMG), stance time, stance time asymmetry index	Locomotor training on a dual-belt instrumented treadmill Baseline, adaptation period and post-adaptation period
Picelli (2015)	RCT-P	30 22 M,8 F	G1: 64.8 ± 6 G2: 61.0 ± 7.2 G3: 62.8 ± 11.8	Chronicity: Chronic Stroke duration (m): G1 (61.3 ± 29.3), G2 (54.8 ± 32.9), G3 (51.9 ± 41.1) Lesion localization: cortical: 11, subcortical: 8, mixed: 11 Side affected: R = NR; L = NR	G1: RAGT + a-tDCS + sham tsDCS G2: RAGT + sham tDCS + c-tsDCS G3: RAGT + a-tDCS + c-tsDCS	Walking capacity (6MWT), walking independence/functional ambulation (FAC), CME, paretic LL strength (MI) muscle tone/spasticity (AS), cadence, S/D support duration ratio	RAGT (motor) + tDCS Pre, post, 2 weeks and 4 weeks follow up
Picelli (2018)	RCT-P	20 13 M, 7 F	G1: 62.60 ± 8.25 G2: 62.80 ± 11.81	Chronicity: Chronic Stroke duration: G1 (67.10 ± 46.75), G2 (51.90 ± 41.15), Lesion localization: cortical: 7, subcortical: 7, mixed: 6 Side affected: R = NR; L = NR	G1: RAGT + c-tcDCS + c-tsDCS G2: RAGT + a-tDCS + c-tsDCS	Walking capacity (6MWT), walking independence/functional ambulation (FAC), CME, paretic LL strength (MI) muscle tone/spasticity, cadence, S/D support duration ratio	RAGT (motor) + tDCS Pre, post, 2 weeks and 4 weeks follow up
Picelli (2019)	RCT-P	40 21 M,19 F	G1: 63.90 ± 10.6 G2: 65.60 ± 9.7	Chronicity: Chronic Stroke duration: G1 (66.4 ± 48.8), G2 (61.70 ± 40.1), Lesion localization: cortical: 14, subcortical: 14, mixed:12 Side affected: R = NR; L = NR	G1: RAGT + contralesional c-tcDCS + c-tsDCS G2: RAGT + Ipsilesional c-tcDCS + c-tsDCS	Walking capacity (6MWT), walking independence/functional ambulation (FAC), CME, paretic LL strength (MI) muscle tone/spasticity, cadence, S/D support duration ratio	RAGT (motor) + tDCS Pre and post
Tani (2025)	RCT-P	20 18 M, 2 F	G1: 56.4 ± 11.7 G2: 54.3 ± 8.0 G3: 49.3 ± 7.7	Chronicity: Chronic Years after onset: G1: 6.9 ± 4.8; G2: 5.7 ± 5.5; G3: 1.6 ± 0.9 Stroke type: IS = 6, HM = 14 Side affected: *R* = 10; L = 10	G1: Spinal stimulation + hip extensor electrical stimulation + treadmill gait training (FAST walk) G2: Spinal stimulation + treadmill gait training (spinal stim) G3: Treadmill gait training (treadmill)	Time for 10-meter walk test, swing time and stance time, time symmetry index (TSI), spinal reciprocal inhibition (assessed with a soleus H reflex conditioning-test paradigm)	Treadmill gait training (motor) + spinal stimulation Before intervention, post intervention and post-4 weeks assessment

*a*-*tDCS:* Anodal transcranial direct current stimulation, *a*-*tsDCS:* Anodic Transcutaneous Spinal Direct Current Stimulation, *BLTT:* Backward locomotor treadmill training, *CCN:* Control condition, *CG:* Control group, *CME:* Corticomotor excitability, *CR:* Crossover, *c*-*tDC: *Cathodal Transcutaneous Spinal Direct Current Stimulation, *DIT:* Dual-belt instrumented treadmill, *ECN:* Experimental condition, *EG*: Experimental group, *F:* Female, *G:* Group, *HM:* Haemorrhagic, *IS:* Ischaemic, *L:* Left, *LL:* Lower limb, *M:* Male, *m:* Months, *N:* Total sample size, *NR:* Not reported, *R:* Right, *RAGT:* Robot assisted gait training, *RCT*-*CR:* Crossover Randomise controlled trial, *RCT*-*P:* Parallel Randomise controlled trial, *SD:* Standard deviation, *S*/*D:* Single/double, *tcDCS:* Cerebellar transcranial direct current stimulation, *c*-*tcDCS:* Cathodal cerebellar transcranial direct current stimulation, *tDCS:* Transcranial direct current stimulation, *tsDCS:* Transcutaneous Spinal Direct Current Stimulation, *tSCS:* Transcutaneous electrical spinal cord stimulation, *TSS:* Transcutaneous spinal stimulation, *VF:* Visual feedback, *6MWT:* Six-minute walk test

#### Reported outcomes

##### Walking function parameters

Walking speed was reported in three studies [12, 24, 25], cadence in four studies [25–28]; walking capacity in five studies [12, 25–28]; functional ambulation/walking independence, paretic lower limb strength, muscle tone/spasticity, and single/double support duration ratio in three studies [26–28]; time symmetry index, swing time, and stance time in one study [24], and stride length was reported in one study [25]. The vertical ground reaction force of the affected lower limb, muscle activity (EMG) from the eight muscles in the affected lower limb, stance time, and stance time asymmetry index were reported in another study [29].

##### Other related parameters

Corticomotor excitability was reported in three studies [26–28], and spinal reciprocal inhibition was reported in one study [24].

### Spinal stimulation protocols

#### Types of stimulation

Cathodal tsDCS was used in three studies [26–28], while anodal tsDCS was used in one study [25]. Cathodal tSCS was used in two studies [12, 24], whereas cathodal phasic TSS was used in another study.

#### Location of electrodes during stimulation

In anodal tsDCS study, the anode was placed on T-11/12 and the reference electrode was placed on the right shoulder [25]. In one tSCS study [12], the electrodes were placed on T11-T12, T11, L1, and C5-C6. In another tSCS study [24], the cathode was placed in the Th11-12 spinal process and the anode on the lower margin of the sternum. In a cathodal phasic TSS study [29], the cathode was placed at the intervertebral space between T12 and L1, while the anode was placed over the anterior iliac crest on the affected side. In all the cathodal tsDCS studies [26–28], the cathode was placed at T10 (T9-T11) and the anode was placed above the unaffected shoulder.

#### Intensity and duration of stimulation

Four of the included studies reported intensities of 2.5mA [25–28]. Other used 20-60mA [12],11–27.5mA [29], and 10.38 ± 2.02mA [24]. The reported durations were 20 min [26–28], 30 min [24, 25], 40 min [12], and 4–6 min [29].

#### Number of sessions

Ten sessions were administered in four studies, and one study each administered six [25], 12 [12], and one session [29].

#### ***Adverse events/effects***

In four studies, no adverse events/effects were recorded/observed, and in one study, no serious adverse events occurred, while two studies were not explicit in reporting information about adverse events/effects. Details are in Table 3.

**Table 3:** Spinal stimulation protocols and findings in the included studies

Studies	Findings						
Author (Year)	Type	Electrode Locations	Intensity (mA) Duration (minutes)	Number of sessions Adverse events/effects	Results/Findings		Conclusion
Awosika (2020)	tsDCS (Anodal)	AN: T-11/12 RF: Right shoulder	2.5 mA 30 min	6 sessions (3 sessions per week) No serious AE	BLTT and tsDCS are well tolerated, feasible and safe techniques for walking rehabilitation training in stroke. Preliminary findings on walking speed and capacity indicate a clinically significant and sustained improvement, lasting at least two weeks, following six sessions of BLTT. The extent of improvement in walking capacity and speed was not greater with anodal tsDCS compared to BLTT combined with sham tsDCS.		Both BLTT and tsDCS were both feasible and safe methods worth further investigation, as likely techniques to optimize post stroke walking recovery.
Moshonkina (2022)	tSCS (Cathodal)	Cathode positions: Midline between T11-T12, T11, L1, and between C5-C6	20–60 mA 40 min	12 daily sessions NR	Following the course of rehabilitation, minimal clinically important differences in walking parameters were attained in the main group, compared to control group.		In rehabilitation, the use of neuro-prosthesis over a 2-week course is enough to achieve minimal clinically important changes in walking parameters.
Park (2025)	Phasic TSS (Cathodal)	CT: Along the midline of the spine at the intervertebral space between T12 and L1 AN: Over the anterior iliac crest on the affected side	11 to 27.5 mA 4–6 min	1 session NR	The changes in vGRF from baseline to the late post-adaptation period were significantly more pronounced in the ECN compared to the CCN. The changes in hip abductor muscle activation and stance time of the affected limb from baseline to early post-adaptation were significantly more pronounced in the ECN compared to the CCN. The changes in stance time asymmetry from the baseline to the early post-adaptation phase exhibited significant differences between the two testing conditions.		Targeted spinal stimulation combined with visually guided weight transfer during locomotor training, can promote improvements in weight transfer and facilitates utilization of the affected limb, potentially achieving symmetrical gait in post-stroke hemiparetic individuals.
Picelli (2015)	tsDCS (Cathodal)	c-tsDCS CT: T10 (T9-T11) AN: above the unaffected shoulder a-tDCS AN: Ipsilesional primary motor area CT: Contralateral above orbit	tsDCS: 2.5 mA tDCS: 2.0 mA 20 min	10 sessions (5 days per week for two weeks) No AE occurred	There was significantly larger improvement in walking capacity and gait cadence at post treatment and 2 weeks follow up assessments in patients that received cathodal tsDCS and anodal tDCS than in those who received cathodal tsDCS or anodal tsDCS singly.		Anodal tDCS combined with thoracic cathodal tsDCS could be beneficial in improving the effects of RAGT in chronic stroke patients.
Picelli (2018)	tsDCS (Cathodal)	c-tsDCS CT: T10 (T9-T11) AN: above the unaffected shoulder tDCS AN: Ipsilesional primary motor area CT: Contralateral above orbit	tsDCS: 2.5 mA tDCS/tcDCS: 2.0 mA 20 min	10 sessions (5 days per week for two weeks) No AE were recorded	There was significantly larger improvement in walking capacity in patients who received cathodal cerebellar stimulation combined with cathodal spinal stimulation than those who received anodal cerebral stimulation combined with cathodal spinal stimulation at first post treatment assessments. But the improvements were not sustained at follow up assessments. There was also larger improvement in gait cadence, and paretic lower limb motricity capacity in patients who received cathodal cerebellar stimulation combined with cathodal spinal stimulation.		Cathodal stimulation of the contralesional cerebellar hemisphere (tcDCS) combined with cathodal spinal stimulation (tsDCS) may be a valuable means of improving the effects of RAGT in chronic ischemic stroke patients.
Picelli (2019)	tsDCS (Cathodal)	c-tsDCS CT: T10 (T9-T11) AN: above the unaffected shoulder c-tcDCS CT: Cerebellar hemisphere (contralesional for group 1 and ipsilesional for group 2) AN: buccinator muscle of the same side	tsDCS: 2.5 mA tcDCS: 2.0 mA 20 min	10 sessions (5 days per week for two weeks) No AE were reported	Both groups show significant improvements in walking capacity at all time points, however, there was no significant between groups differences in walking capacity at the first post assessment and follow up period.		Cathodal tDCS over the contralesional or ipsilesional cerebellar hemisphere in combination with cathodal tsDCS might lead to similar effects on RAGT in chronic supratentorial stroke patients.
Tani (2025)	tSCS (Cathodal)	CT: Th11-12 spinal process AN: lower margin of the sternum	Mean stimulation intensity: 10.38 ± 2.02 mA. The Intensity of stimulation was set at twice the sensory threshold. 30 min (two sets of 15 min)	10 sessions (twice weekly for 5 weeks) No AE related to the intervention were observed	The three group’s mean improvement in gait speed was greater than the minimal clinical important difference but not significantly different across the groups, however, spinal stimulation + hip extensor electrical stimulation + treadmill gait training (FAST walk) group had a significant improvement in gait speed, but not spinal stimulation + treadmill gait training (spinal stim) group and treadmill gait training (treadmill) group. Across the three groups, there were no significant changes in spinal reciprocal inhibition.		The recently developed FAST walk, which utilise transcutaneous spinal cord and hip stimulation driven by electromyography, is safe and could improve the gait speed of individuals with chronic stroke.

*AN:* Anode electrode, *a*-*tDCS:* Anodal transcranial direct current stimulation, *BLTT:* Backward locomotor treadmill training, *CCN:* Control condition, *CT:* Cathode electrode, *ECN:* Experimental condition, *EEG:* Electroencephalogram, *G:* group, *mA:* milliampere, *No:* No adverse effect reported by participants, *NR:* No information reported in the study, *RAGT:* Robot assisted gait training, *RF:* Reference electrode, *tcDCS:* cerebellar transcranial direct current stimulation, *c*-*tcDCS:* Cathodal cerebellar transcranial direct current stimulation, *tDCS:* Transcranial direct current stimulation, *TSS:* Transcutaneous spinal stimulation, *c*-*tsDCS:* Cathodal Transcutaneous Spinal Direct Current Stimulation, *tsDCS:* Transcutaneous Spinal Direct Current Stimulation, *tSCS:* Transcutaneous electrical spinal cord stimulation, *TT:* treadmill training, *vGRF:* Vertical ground reaction force

## Narrative synthesis

### Effect of the interventions

#### Effects on walking function parameters

##### Walking capacity

Significant larger improvements in walking capacity were reported when gait training was combined with cathodal tsDCS and anodal tDCS [28] and with cathodal spinal stimulation and cathodal cerebellar stimulation [27]. Furthermore, the main intervention group showed greater walking capacity compared to the control group, achieving a statistically significant improvement that exceeded the MCID by approximately 30%. The change in the control group was not significant and did not meet the clinical benchmarks [12]. Additionally, even though both groups demonstrated significant improvements in walking capacity at all time points, no significant between-group differences were found when gait training was combined with cathodal tsDCS and contralesional cathodal transcranial direct current stimulation of the cerebellum (tcDCS) compared with when it was combined with cathodal tsDCS and ipsilesional cathodal tcDCS [26]. However, no significant between-group differences in improvement in walking capacity were observed when gait training was combined with anodal tsDCS compared to when it was combined with sham stimulation [25].

##### Cadence

Significant larger improvements in gait cadence were reported when gait training was combined with cathodal tsDCS and anodal tDCS [28] and with cathodal spinal stimulation and cathodal cerebellar stimulation [27]. Additionally, in another study [26] both the two groups (contralesional and ipsilesional) demonstrated significant improvements in cadence within their respective groups; however, no significant differences were observed between the two groups. According to an anodal tsDCS study [25], both groups showed improved cadence during the 10 MWT, and sham tsDCS participants had greater cadence improvement from baseline to post-intervention, though this was insignificant after adjusting for baseline walking speed. Both groups maintained cadence gains for two weeks post-training, with no group differences from post-intervention to the 2-week follow-up.

##### Paretic lower limb strength

Gait training combined with cathodal spinal stimulation and cathodal cerebellar stimulation showed significantly greater improvement in paretic lower limb strength [27]. However, two other studies [26, 28] did not observe any significant difference in lower limb muscle strength between the groups.

##### Walking speed

Based on a cathodal tSCS study [12], the comfortable walking speed within the main group exhibited a notable increase, whereas no change was observed in the control group. This improvement in comfortable walking speed for the main group was significant and exceeded the MCID, compared to the very minimal/little or no change in the control group. Additionally, in another cathodal tSCS study [24], the FAST walk group showed significant improvement in 10-meter walking time after the intervention and four weeks after the end of the intervention. However, the other groups showed no significant improvements at these time points compared to baseline, and no significant difference in walking time was observed between the groups. In anodal tsDCS study [25], both groups showed significant walking speed improvement on 10 MWT after BLTT, reaching MCID for stroke recovery. Sham tsDCS participants showed greater speed improvement from baseline to post-intervention compared to anodal tsDCS, although this difference became non-significant after baseline differences in walking speed adjustment.

##### Secondary gait outcomes and related parameters

Statistically significant improvements within-group were observed in the single/double support duration ratio [26–28], and stride length [12], with a numerical improvement observed in the time symmetry index for the treadmill-only group [24]. However, no significant differences were observed between groups in terms of walking independence, muscle tone, and single/double support duration ratio [26–28], as well as in time symmetry index and spinal reciprocal inhibition [24], and stride length [25]. The only crossover study [29] reported that compared to sham stimulation, the active stimulation condition resulted in significantly greater improvements in paretic hip abductor activation, paretic lower limb stance time, and stance time symmetry during early post-adaptation. Additionally, weight transfer improved significantly in the late post-adaptation phase.

#### Synthesis of findings

Overall, cathodal transcutaneous spinal stimulation combined with gait training probably improves primary gait outcomes with moderate certainty, while exhibiting little or no difference in secondary gait outcomes. In contrast, anodal tsDCS showed variable/mixed effects on gait outcomes based on evidence from a single moderate-certainty study. The Summary of Findings (SoF) table (Table 4) provides a detailed account of the findings.

**Table 4:** Summary of findings for gait-related outcomes in stroke survivors

Outcome	Studies (*n*)	Effect/Narrative statement	Certainty
Waking capacity assessed with 6MWT (cathodal studies)	4 RCTs (110)	Consistent positive effects/improvements in all (3 sig between-group; 1 withing-group only)	Moderate ⊕⊕⊕⊝ ↓1 Imprecision
Waking capacity assessed with 6MWT (anodal study)	1 RCT (30)	Both groups reached MCID (34.4); *P* < 0.001; anodal tsDCS: +41.96 m vs. sham + 92 m Sham greater: unadj. *P* = 0.050, adj. *P* = 0.082	Moderate ⊕⊕⊕⊝ ↓1 Imprecision
Walking speed assessed with 10MWT (cathodal studies)	2 RCTs (40)	Inconsistent effects (1 study significant benefit in main group vs. control, *P* = 0.013, > MCID; another study within-group improvement: *P* = 0.024, no between-group difference: *P* = 0.578)	Low ⊕⊕⊝⊝ ↓1 Imprecision, ↓1ROB
Walking speed assessed with 10MWT (anodal study)	1 RCT (30)	Both groups > MCID (0.16 m/s, *P* < 0.001 each); anodal + 0.215 vs. sham + 0.412; sham greater: unadj. *P* = 0.016, adj. *P* = 0.054	Moderate ⊕⊕⊕⊝ ↓1 Imprecision
Cadence as steps or cycles per minute (cathodal studies)	3 RCTs (90)	Consistent positive effects/improvements in all (2 sig between-group; 1 withing-group only)	Moderate ⊕⊕⊕⊝ ↓1 Imprecision
Cadence as steps or cycles per minute (anodal study)	1 RCT (30)	Both groups *P* < 0.001; sham greater: unadj. *P* = 0.046, adj. *P* = 0.091; gains maintained at 2 weeks	Moderate ⊕⊕⊕⊝ ↓1 Imprecision
Gait symmetry/time symmetry index (cathodal study)	1 RCT (20)	No significant group x time interaction (*P* > 0.05)	Low ⊕⊕⊝⊝ ↓1 Imprecision, ↓1ROB
Single/double support duration ratio (gait stability) (cathodal study)	3 RCTs (90)	No significant between group differences in all (*P* > 0.05)	Moderate ⊕⊕⊕⊝ ↓1 Imprecision
Stride length (anodal study)	1 RCT (30)	Both groups sig. *P* < 0.001; no significant between-group diff.; gains maintained at 2 weeks; clinical meaningful improvements	Moderate ⊕⊕⊕⊝ ↓1 Imprecision
Paretic lower limb strength assessed with Motricity index (cathodal studies)	3 RCTs (90)	Inconsistent effects (1 study significant between-group difference/benefit at all timepoints *P* ≤ 0.045; 2 studies no significant between-group differences)	Moderate ⊕⊕⊕⊝ ↓1 Imprecision
Walking independence assessed with FAC (cathodal studies)	3 RCTs (90)	No significant between group differences in all (*P* > 0.05)	Moderate ⊕⊕⊕⊝ ↓1 Imprecision
Muscle tone/spasticity assessed with MAS (cathodal studies)	3 RCTs (90)	No significant between group differences in all (*P* > 0.05)	Moderate ⊕⊕⊕⊝ ↓1 Imprecision
Spinal reciprocal inhibition (cathodal study)	1 RCT (20)	No significant group x time interaction (*P* > 0.05)	Low ⊕⊕⊝⊝ ↓1 Imprecision, ↓1ROB
vGRF, muscle activity, stance time and stance time asymmetry index (cathodal study)	1 crossover RCT (11)	Significant improvements across all the 4 outcomes (*P* < 0.05).	Low ⊕⊕⊝⊝ ↓1 Imprecision, ↓1ROB
Overall gait outcomes (cathodal studies)	6 RCTs (5 PR, 1 CR; *n* = 171)	Cathodal transcutaneous spinal stimulation with gait training probably improves primary gait outcomes (directionally supported by 1 crossover RCT); consistent benefits/positive direction across walking capacity (4 RCTS), cadence (3 RCTS), paretic lower limb strength (3 RCTS), and walking speed (2 RCTS). The cathodal stimulation with gait training probably had little to no effects on secondary gait outcomes; consistent null between-group differences across time symmetry index, single/double support duration ratio, walking independence, muscle tone and spinal reciprocal inhibition.	Moderate ⊕⊕⊕⊝ ↓1 Imprecision
Gait outcomes (anodal study)	1 parallel RCT (30)	Anodal transcutaneous spinal stimulation with gait training may make little or no difference in improving gait outcomes compared with sham (Mixed: both groups MCID walking capacity/walking speed, both sig. cadence/stride length; sham greater in ¾ of outcomes, no sig. between-group diff in ¼ of outcome; gains maintained at 2 weeks in 2/4 of outcomes).	Moderate ⊕⊕⊕⊝ ↓1 Imprecision

*6* *MWT:* Six-minute Walk Test, *10MWT:* Ten-meter Walk Test, *FAC:* Functional Ambulation Category, *MAS:* Modified Ashworth Scale, *vGRF:* Vertical Ground Reaction Force, *PR:* Parallel, *CR:* Crossover, *unadj:* unadjusted, *adj:* adjusted

Comparators: sham spinal stimulation/active variant (*n* = 4  sham-active, including I crossover RCT, *n* = 2 active-active RCTs); gait training alone (*n* = 1 RCT). Table 2 presents the details

Intervention: Gait training + active spinal stimulation (alone or with adjuvant stimulation)

Comparison: Gait training + sham spinal stimulation/active variant (alone or with adjuvant stimulation) or gait training alone

##### Intervention

Gait training + active spinal stimulation (alone or with adjuvant stimulation).

##### Comparison

Gait training + sham spinal stimulation/active variant (alone or with adjuvant stimulation) or gait training alone.

## Discussion

The key objective of this study was to determine the scientific evidence supporting the effect of TSS combined with gait training on walking-related outcomes in stroke survivors. The main findings from the qualitative synthesis indicate consistent benefits of gait training combined with cathodal TSS on walking capacity, cadence, walking speed, and paretic lower limb strength, supported by moderate-certainty evidence in stroke survivors. It is crucial to focus on and prioritise approaches for improving gait in stroke survivors, as achieving ambulatory independence is a primary objective for patients undergoing post-stroke rehabilitation [6]. The review included seven RCTs (six parallel and one crossover), and qualitative synthesis was used for data analysis.

### Qualitative findings

The qualitative synthesis findings indicated that gait training combined with cathodal TSS likely improves walking capacity, cadence, walking speed, and paretic lower limb strength in stroke survivors (moderate-certainty evidence). These improvements may be associated with the integration of supraspinal and spinal mechanisms that govern the neural regulation of locomotion [28]. Another possible contributing factor is the use of gait training, especially treadmill and robot-assisted gait training, used in the studies. These improvements in gait outcomes are crucial because, in addition to independence, speed, and endurance, stroke survivors also emphasise the significance of walking quality [30]. However, the evidence for some of these improvements is limited by the findings and conclusions based on the qualitative synthesis of the included studies. Therefore, further research is needed to enable more robust meta-analyses to confirm these findings.

Positive effects on walking capacity, cadence, and muscle strength in the paretic lower limb were predominantly observed in studies involving the combination of cathodal tsDCS with RAGT. This highlights the significance of the combined approach. The positive effects of tsDCS likely align with a previous study reporting that the observed positive effects of tsDCS may be attributed to the locomotor system modulation induced by tsDCS, potentially through its impact on spinal pathways and neuronal connectivity [31]. The contributory impact of RAGT might align with a previous study [31], which suggested that favourable outcomes could be attributed to the low-intensity characteristics of RAGT, which was optimised to facilitate motor learning. A positive effect on walking speed was observed in two studies, one on cathodal tSCS combined with treadmill gait training and the other on cathodal tSCS combined with walking/stepping on a treadmill and floor. This positive impact may align with a previous review on tSCS [32], which noted that stroke survivors and individuals with multiple sclerosis and spinal cord injury exhibited notably improved walking speed, symmetry, and stride length, suggesting that tSCS enhances motor learning by facilitating neuroplasticity and mitigating spasticity.

Additionally, while improvements in primary walking function parameters have been reported, most studies showed significant improvements within groups across the three outcomes. Notably, between-group differences were observed in most studies for walking capacity and cadence, but in a few studies for paretic lower limb strength. These inconsistencies underscore the need for further RCTs to draw definitive conclusions. One potential explanation for the non-significant between-group differences is that in two studies, the comparative groups underwent active interventions that varied only in tDCS add-on placement, specifically contralateral versus ipsilateral cerebellar positioning or cathodal cerebellar versus anodal cerebral site.

Moreover, in one of the studies [12], the main intervention group showed significant improvement in comfortable walking speed beyond the MCID, while the control group showed minimal change, demonstrating the beneficial role of cathodal tSCS. Another study [24] showed that cathodal tSCS + hip extensor electrical stimulation + treadmill gait training led to significant improvements in gait speed. Although the findings demonstrated non-significant superiority of the intervention over other intervention groups, it established how gait speed was significantly improved in the EMG-driven transcutaneous spinal stimulation group but not in other groups, thus showing the potential of the intervention in improving gait speed. Thus, future studies with more methodological rigor are needed to further explore the effects of these interventions. Improvement in walking speed is instrumental in stroke gait, as walking speed and gait symmetry are primary metrics utilised in the assessment of walking recovery [33].

Furthermore, the significantly larger improvements observed in the active stimulation condition for paretic hip abductor activation, paretic lower limb stance time, and symmetry in the crossover study (cathodal phasic TSS + VF  + locomotor training) further corroborate the primary findings related to walking function outcomes. Conversely, cathodal TSS exhibited little or no difference in secondary gait outcomes, including symmetry index, single/double support duration ratio, walking independence, muscle tone, and spinal reciprocal inhibition. Anodal tsDCS shows variable/mixed effects across gait outcomes. While it achieves the MCID for certain outcomes and significantly improves others, in some cases, sham stimulation results in greater improvements, and in other instances, no significant between-group differences are observed. The observed effects of cathodal TSS and anodal tsDCS may be attributed to the limited number of studies that have investigated certain outcomes and the variability present in the comparison groups of some studies. Therefore, additional research is necessary to further investigate the evidence for these outcomes.

Additional factors that may have influenced the findings of this review include the specific type of gait treatment employed, nature of the comparisons made, and dosage of the treatment administered. In particular, three studies focused on robot-assisted gait training, three on treadmill gait training, and one on both treadmill and overground trainings. For comparisons, studies have investigated gait training combined with active spinal stimulation versus sham stimulation, with or without additional therapy. Other studies have compared groups receiving active spinal stimulation combined with gait training, differing only in the additional stimulation type. Additionally, none of the studies included more than 12 treatment sessions, with most implementing 10 treatment sessions. Such factors and/or variabilities may have influenced the outcomes of this review. Furthermore, the chronic stage characteristics of the stroke survivors who participated in all the studies may have influenced the findings. Thus, these factors should be considered when designing future studies.

The studies included in the review were of good or excellent quality; however, some lacked adequate data on outcomes, which prevented meta-analysis from being conducted for those outcomes. Therefore, we relied on the qualitative synthesis of these outcomes. Additionally, the included studies had limited sample sizes, and some exhibited an unclear ROB in random sequence generation and performance bias or a high ROB in selection and performance bias. These methodological limitations may affect the strength of the conclusions, highlighting the need for further research to address these issues and provide more robust and definitive findings on this topic.

### Strengths and limitations

The major strengths of this review include adherence to PRISMA guidelines, adherence to Cochrane/GRADE guidelines utilising SoF tables for qualitative synthesis, a comprehensive search of appropriate databases, and the use of suitable evaluation tools to assess the MQ and ROB. However, a key limitation of this review is that a meta-analysis was not conducted because of inadequate data and requirements to conduct the meta-analysis in the studies. Therefore, future research is required to address these gaps. Some of the crucial limitations of the studies included in this review are the limited sample sizes and susceptibility to selection and performance bias in some studies. Consequently, the findings of this review should be interpreted with these limitations in mind. Future studies should address these limitations to strengthen the evidence base.

## Conclusions

Moderate-certainty evidence shows that cathodal transcutaneous spinal stimulation combined with gait training probably improves primary walking outcomes in stroke survivors.

## Supplementary Information

Below is the link to the electronic supplementary material.

ESM 1 Details of the search strategy employed in most of the online data bases. (PDF 8.64 KB)
