# Supplementary material for: Effects of transcutaneous spinal stimulation with gait training on walking-related outcomes in stroke survivors: a systematic review
Source: Neurol Sci. 2026 Jan 31;47(2):213. doi: 10.1007/s10072-026-08822-x (PMC12858533; doi:10.1007/s10072-026-08822-x)
Supplement: Supplementary file 1 — Details of the search strategy employed in most of the online data bases. (PDF 8.64 KB) [file 10072_2026_8822_MOESM1_ESM.pdf]

Supplementary Material: Search strategy used in PubMed database, and similar strategy was used in other databases

((("Transcutaneous spinal stimulation"[All Fields] OR "Transcutaneous spinal cord stimulation"[All Fields]) AND "Transcutaneous spinal direct current stimulation"[All Fields]) OR "Transpinal direct current stimulation"[All Fields] OR "spinal cord stimulation"[All Fields]) AND ("Stroke"[MeSH Terms] OR "Cerebrovascular Disorders"[MeSH Terms] OR "Hemiplegia"[MeSH Terms] OR "Paresis"[MeSH Terms] OR ("Stroke"[MeSH Terms] OR "Stroke"[All Fields] OR "strokes"[All Fields] OR "stroke s"[All Fields] OR ("Hemiplegia"[MeSH Terms] OR "Hemiplegia"[All Fields] OR "hemiplegias"[All Fields]) OR ("Paresis"[MeSH Terms] OR "Paresis"[All Fields] OR "hemiparesis"[All Fields]) OR "cerebrovascular accident"[All Fields] OR ("disease"[MeSH Terms] OR "disease"[All Fields] OR "diseases"[All Fields] OR "disease s"[All Fields] OR "diseased"[All Fields]))) AND ("Walking"[MeSH Terms] OR "Walking Speed"[MeSH Terms] OR "Mobility Limitation"[MeSH Terms] OR "Motor Activity"[MeSH Terms] OR "Lower Extremity"[MeSH Terms] OR ("gait"[MeSH Terms] OR "gait"[All Fields] OR ("walked"[All Fields] OR "Walking"[MeSH Terms] OR "Walking"[All Fields] OR "walks"[All Fields]) OR ("mobilities"[All Fields] OR "range of motion, articular"[MeSH Terms] OR ("range"[All Fields] AND "motion"[All Fields] AND "articular"[All Fields]) OR "articular range of motion"[All Fields] OR "mobility"[All Fields]) OR "motor function"[All Fields] OR "lower extremity function"[All Fields]))
